# Therapeutic Effects of *Acer palmatum* Thumb. Leaf Extract (KIOM-2015E) on Benzalkonium Chloride-Induced Dry Eye in a Mouse Model

**DOI:** 10.3390/ijms232314964

**Published:** 2022-11-29

**Authors:** Nam-Hui Yim, Eunhee Park, Won-Kyung Cho, Yeoun-Hee Kim, Jin Yeul Ma

**Affiliations:** 1 Korean Medicine (KM)-Application Center, Korea Institute of Oriental Medicine (KIOM), 70, Cheomdan-ro, Dong-gu, Daegu 41062, Republic of Korea; 2 Helixmith, 21, Magokjungang 8-ro 7-gil, Gangseo-gu, Seoul 07794, Republic of Korea; 3 R&D Center, Etnova Therapeutics Corp., 198, Saneop-ro, Gwonseon-gu, Suwon-si 13207, Republic of Korea

**Keywords:** benzalkonium chloride, dry eye syndrome, inflammation, apoptosis, natural agents

## Abstract

We determined the effects of two extracts from *Acer palmatum* Thumb. leaves (hot water extract KIOM-2015EW and 25% ethanol extract KIOM-2015EE) in a benzalkonium chloride (BAC)-induced dry eye mouse model. Dry eye was induced by 0.2% BAC for 2 weeks, followed by treatment three times (eye drop) or once (oral administration) daily with KIOM-2015E for 2 weeks. Treatment with both KIOM-2015EE and KIOM-2015EW resulted in a marked increase in tear volume production for the 4 days of treatment. The Lissamine Green staining score, TUNEL-positive cells, and inflammatory index were significantly decreased after 2 weeks. Topical KIOM-2015EE administration exhibited a greater improvement in decreasing the ocular surface staining scores, inflammation, dead cells, and increasing tear production in a dose-dependent manner compared with the other groups. Furthermore, KIOM-2015E significantly reduced the phosphorylation of NF-κB, which was activated in the BAC-treated cornea. Topical administration was much more effective than oral administration for KIOM-2015E and KIOM-2015EE was more effective than KIOM-2015EW. Application of KIOM-2015E resulted in clinical improvement, inhibited the inflammatory response, and alleviated signs of dry eye. These results indicate that KIOM-2015E has potential as a therapeutic agent for the clinical treatment of dry eye.

## 1. Introduction

Dry eye is a common disease of the tear and ocular surface that affects millions of people worldwide. Although the causes are multifactorial, it results in symptoms of ocular discomfort, visual disturbance, and an instability of tear films [1,2]. This is accompanied by increased osmolarity of the tear film and inflammation of the ocular surface [2]. Disease or dysfunction of the tear glands results in changes in tear composition, such as hyperosmolarity, which stimulate the production of inflammatory mediators on the ocular surface [2,3].

Recent studies have identified side effects caused by preservatives, such as BAC [4]. The toxicity of BAC to the ocular surface has been examined in several in vivo experiments by administering BAC to mice [5], rats [6], and rabbits [7]. These BAC-induced dry eye models share the same pathologic changes associated with human dry eye, including epithelial apoptosis, goblet cell loss, tear film defect, and inflammation, which were described in previous reports [8,9]. BAC accumulation induces a reduction in mucins and alteration of the lipid layer, resulting in impairment of the tear film with tear instability and excessive evaporation, which are hallmarks of dry eye disease [10].

Inflammation may, in turn, cause dysfunction or disappearance of the cells responsible for tear secretion or retention [11]. Increased production and activation of pro-inflammatory cytokines, such as interleukin (IL)-1 and tumor necrosis factor (TNF)-α, and proteolytic enzymes by the stressed ocular surface and glandular epithelial cells, as well as by the inflammatory cells that infiltrate these tissues, occur in dry eye [12,13]. Increased concentrations of pro-inflammatory cytokines and chemokines in the tear fluid, such as IL-6, IL-1, and TNF-α, have also been reported [11,14,15,16]. Experimental dryness significantly increases the expression of these transcripts in the corneal epithelium and conjunctiva of C57BL/6 mice [17]. Therefore, anti-inflammatory therapy has become a common strategy for treating dry eye disease.

The majority of anti-inflammatory agents currently in use to treat dry eye include topical corticosteroids and immunomodulatory agents. Corticosteroids are potent anti-inflammatory agents that are routinely used to control inflammation in various organs. They have been used successfully to treat corneal epithelial disease in dry eyes [18,19]; however, because of the possible serious side effects of prolonged use, the use of steroids such as Fluoremetholone (FML) for chronic therapy of dry eye may be limited [2]. Cyclosporine A (CsA) has shown the greatest efficacy for moderate-to-severe dry eye patients [2,20]; however, it remains to be determined whether CsA therapy is beneficial for long-term treatment of dry eye. Thus, further studies are necessary to delineate the appropriate time course of CsA therapy required to achieve lasting effects [20]. Moreover, CsA is not available in all countries as its use has not been approved worldwide. Therefore, new therapeutics for dry eye therapy that are more effective and reliable are needed.

KIOM-2015E is a hot water (KIOM-2015EW) or 25% EtOH (KIOM-2015EE) extract from *Acer palmatum* Thumb. The roots, bark, twigs, and leaves of this plant has been used in traditional Chinese medicine for alleviating pain, detoxification, relaxing muscles, and stimulating blood circulation [21]; however, its use for the treatment of dry eye syndrome and its anti-inflammatory effects have not been fully evaluated. Recently, we demonstrated that KIOM-2015EW has anti-inflammatory and anti-apoptotic effects in a hyperosmolar, stress-induced in vitro dry eye model [22]. In the present study, we determined the effect of KIOM-2015E in treating dry eye disease in vivo using a BAC-induced dry eye mouse model.

## 2. Results

### 2.1. KIOM-2015E Ameliorates Corneal Dysfunction in a BAC-Induced Dry Eye Mouse Model

**Figure 1:**
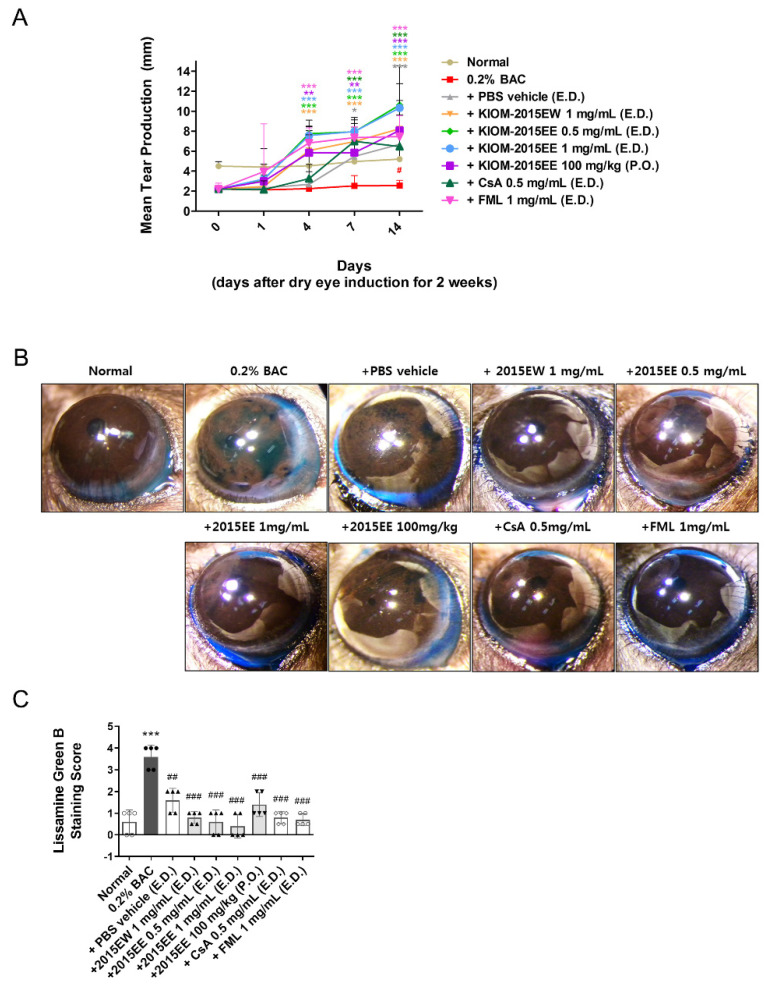
KIOM-2015E improves aqueous tear production and corneal epithelial damage in a BAC-induced dry eye model (n = 5/group). Alterations of the ocular surface in were observed in clinical evaluations on days 0, 1, 4, 7, and 14 for each group. The normal group was left untreated. The BAC group was also treated with 0.2% BAC for the remaining 2 weeks. Eye drops were administered three times a day for the specified time, whereas oral administration was carried out once a day. (**A**) The mice were anesthetized and Zone-Quick phenol red thread cotton was applied to the lower eyelid palpebral conjunctiva to absorb tears for 60 s. (**B**) Representative images of the corneal epithelium integrity, including Lissamine Green B staining on day 14 after KIOM-2015E application. (**C**) Changes in the Lissamine Green B staining score relative to treatment in KIOM-2015E-treated eyes at 2 weeks. Data were analyzed for statistical significance using an analysis of variance followed by a two-way ANOVA for multiple comparisons using GraphPad Prism software. Differences were considered statistically significant as follows: # *p* < 0.05 vs. Normal, * *p* < 0.05, ** *p* < 0.01, *** *p* < 0.001 vs. BAC. Each color of *p* value corresponds to the color of each administration group. The “0D” indicates the measurement 14 days after BAC application in (**A**). *** *p* < 0.001 vs. Normal, ^##^ *p* < 0.01, ^###^ *p* < 0.001 vs. BAC in (**C**). E.D.; topical eye drop, P.O.; oral administration; 2015EW; KIOM-2015EW (hot water extraction), 2015EE; KIOM-2015EE (25% EtOH extraction), CsA; cyclosporine, FML; Fluorometholone. White circle + white bar, Normal; dark gray circle + dark gray bar, 0.2% BAC; Dark gray triangle + white bar, PBS vehicle; Dark gray triangle + light gray bar, 2015EW and 2015EE eye drop; Dark gray inverted triangle + light gray bar, oral administration of 2015EE; White diamond + white bar, CsA; White Gakjinwon + White Bar, FML.

To determine the effects of KIOM-2015E on corneal dysfunction in dry eye mice, KIOM-2015E was administered daily at doses of 0.5 and 1 mg/mL topically to the corneas or 100 mg/kg orally to BAC-induced dry eye mice (Supplementary Figure S1). Clinical evaluations, including Schirmer’s test and corneal Lissamine Green B staining, were conducted (Figure 1, Supplementary Figures S2 and S5A,B).

On days 0, 1, 4, 7, and 14 after treatment with KIOM-2015E, five mice were randomly selected from each group. Anesthesia was performed and tear volume was measured. Using a phenol red cotton thread, we measured the length of the area changed to red by a tear (Figure 1A). After 2 weeks of KIOM-2015E treatment, anesthesia was performed. Then, 1% Lissamine Green B was applied topically to the eye and the surface was photographed with a digital camera (Figure 1B). Before BAC administration, there was no statistically significant difference in aqueous tear production or corneal staining with Lissamine Green B between the groups. Aqueous tear production was significantly decreased (Figure 1A and Supplementary Figure S5A) and the corneal staining score was increased (Figure 1B,C and Supplementary Figure S5B) on day 14 after administration of BAC compared with the untreated control group (Supplementary Figure S2).

Figure 1A shows the effects of KIOM-2015E on tear production. As shown in the bar graph at day 0, tear production from BAC (2.61 mm ± 0.76) was significantly decreased compared with the normal group (4.5 mm ± 0.51) prior to KIOM-2015E treatment and after 2 weeks of BAC treatment (*p* < 0.01) (Supplementary Figure S5A). After 1 day of drug treatment, tear production increased in all groups except for the CsA- and PBS-treated groups, but there was no statistical significance. However, after 4 days of drug treatment, the PBS vehicle- and CsA-treated groups exhibited slightly increased tear production compared with the BAC group, and tear production was significantly increased in all other groups compared with the normal group. After 1 week of KIOM-2015E treatment, tear production was markedly increased in all groups. Interestingly, tear production increased in a dose-dependent manner for the ethanol extract, but not for the hot water extract. In addition, the effect of KIOM-2015E was clearly confirmed by topical eye drops or oral administration. 

BAC-treated mice showed progressive epithelial damage to the ocular surface and increased Lissamine Green B staining. To assess whether KIOM-2015E could protect the ocular surface epithelium from damage induced by aqueous tear deficiency in BAC-induced dry eye mice, we quantified Lissamine Green B staining in the eyes following treatment with KIOM-2015E, normal saline, and two positive control drugs (CsA and FML). The ocular staining score was significantly decreased after KIOM-2015E treatment compared with pretreatment (Figure 1B,C and Supplementary Figure S5B), which indicated that KIOM-2015E promoted the healing of the corneal epithelial defects. These results also indicate that KIOM-2015E topical eye drops were more effective than oral administration.

### 2.2. KIOM-2015E Application Reduces Inflammatory Cytokine Expression on the Ocular Surface in a BAC-Induced Dry Eye Mouse Model

**Figure 2:**
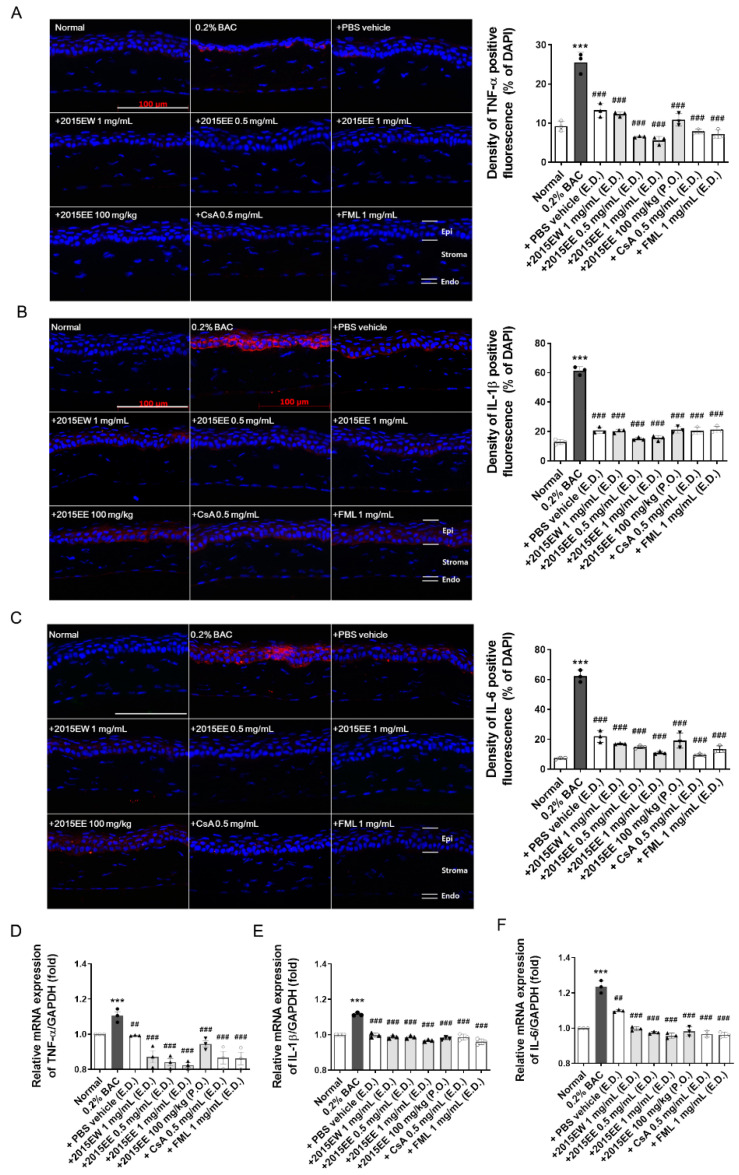
Representative images for immunofluorescent staining and the mRNA expression of TNF-α, IL-1β, and IL-6 to determine corneal epithelial inflammation in a BAC-induced dry eye mouse model. Representative images for corneal cytokine immunolabeling merged with DAPI (blue) and the density of cells positively stained in the cornea tissue were taken. Immunofluorescence staining was performed using TNF-α-specific antibody (red; (**A**)), IL-1β-specific antibody (red; (**B**)), and IL-6-specific antibody (red; (**C**)) in corneal tissues. To quantify the results, bar charts for the relevant images are shown (bar chart in the right panel). Quantification of red-fluorescence density was performed using ImageJ software. The experiments were performed in triplicate and the results represent the average of three independent experiments. The results of the bar graph also represent the averages of three independent experiments. Quantitative RT-PCR analysis of TNF-α, IL-1β, and IL-6 in the BAC-induced mouse ocular surface. After 14 days of KIOM-2015E treatment, the corneas were harvested and analyzed by qRT-PCR to measure the mRNA levels of (**D**) TNF- α, (**E**) IL-1β, and (**F**) IL-6. The data were analyzed for statistical significance using an analysis of variance, followed by a two-way ANOVA for multiple comparisons using GraphPad Prism software. Differences were considered statistically significant as follows: *** *p* < 0.001 vs. Normal, ^##^ *p* < 0.01, ^###^ *p* < 0.001 vs. BAC. E.D.; topical eye drop, P.O.; oral administration; 2015EW; KIOM-2015EW (hot water extraction), 2015EE; KIOM-2015EE (25% EtOH extraction), CsA; cyclosporine, FML; Fluorometholone. Scale bar, 100 µm. Endo, endothelium; Epi, epithelium; Stroma, corneal stroma. White circle + white bar, Normal; dark gray circle + dark gray bar, 0.2% BAC; Dark gray triangle + white bar, PBS vehicle; Dark gray triangle + light gray bar, 2015EW and 2015EE eye drop; Dark gray inverted triangle + light gray bar, oral administration of 2015EE; White diamond + white bar, CsA; White Gakjinwon + White Bar, FML.

To determine the distribution and expression levels of inflammatory marker proteins after exposure to KIOM-2015E, immunofluorescence staining was carried out with specific antibodies against TNF-α, IL-1β, and IL-6 (Figure 2A–C and Supplementary Figure S3 and S5C). After 2-weeks of KIOM-2015E treatment, the eye balls were harvested, fixed, and paraffin-embedded. Cornea tissue sections were prepared, deparaffinized, rehydrated with ethanol, and analyzed by immunofluorescence staining. As expected, strong positive signals for TNF-α (Figure 2A), IL-1β (Figure 2B), and IL-6 (Figure 2C) were observed in both the corneal epithelium and endothelium of the BAC-treated groups. Compared with BAC-treated mice, the staining density for all marker proteins was markedly decreased in the corneas of KIOM-2015E-treated mice. To quantify these results, bar charts for the relevant images are presented in Figure 2. Quantitation of the fluorescence intensity revealed that the expression of BAC-induced inflammatory marker proteins was markedly increased in BAC-treated mice, but KIOM-2015E-treated corneas exhibited a significant decrease. We examined the inhibitory effects of KIOM-2015E on the expression of TNF-α, IL-1β, and IL-6 mRNA (Figure 2D–F and Supplementary Figure S5D). Cornea tissue was isolated from the harvested eye balls. Total RNA was extracted and cDNA was synthesized and analyzed by real-time reverse transcription polymerase chain reaction (RT-PCR). Consistent with the results of immunofluorescence staining, the inflammatory marker mRNAs were increased by BAC treatment and decreased following KIOM-2015E treatment.

### 2.3. KIOM-2015E Application Reduces Damage to the Cornea on the Ocular Surface in a BAC-Induced Dry Eye Mouse Model

**Figure 3:**
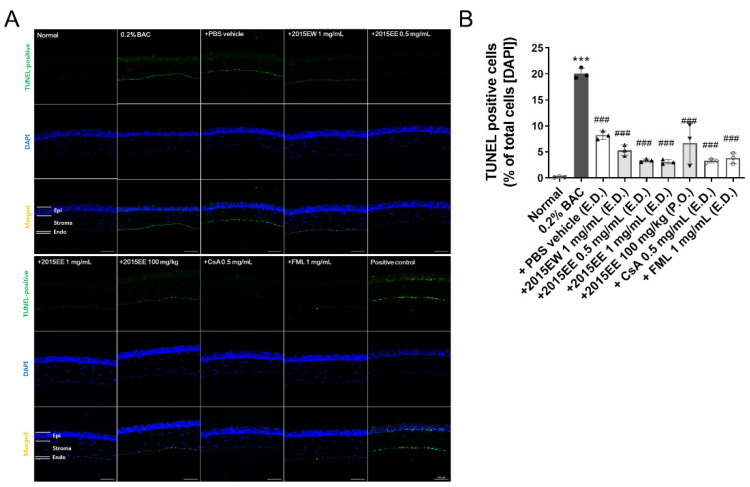
Representative image showing the distribution of apoptotic cells in the corneal tissue treated with 0.2% BAC and/or KIOM-2015E for 2 weeks. A TUNEL assay was used to detect apoptotic nuclei. (**A**), Normal saline, 0.2% BAC, 1 mg/mL KIOM-2015EW (E.D.), 0.5 mg/mL and 1 mg/mL KIOM-2015EE (E.D.), 100 mg/kg KIOM-2015EE (P.O.), 0.5 mg/mL CsA (E.D.), 1 mg/mL FML (E.D.), and positive control. TUNEL staining revealed apoptotic cells following BAC treatment. KIOM-2015E treatment significantly reduced the number of apoptotic cells. (**B**), Mean superficial number of apoptotic cells is shown. All experiments were performed in triplicate. The results in the bar graph are the average of 3 independent experiments. *** p < 0.001 ver. healthy control and *^###^ p* < 0.001 ver. 0.2% BAC. E.D.; topical eye drop, P.O.; oral administration, 2015EW; KIOM-2015EW (hot water extraction), 2015EE; KIOM-2015EE (25% EtOH extraction), CsA; cyclosporine, FML; Fluorometholone. Scale bar, 100 µm. Endo, endothelium; Epi, epithelium; Stroma, corneal stroma. White circle + white bar, Normal; dark gray circle + dark gray bar, 0.2% BAC; Dark gray triangle + white bar, PBS vehicle; Dark gray triangle + light gray bar, 2015EW and 2015EE eye drop; Dark gray inverted triangle + light gray bar, oral administration of 2015EE; White diamond + white bar, CsA; White Gakjinwon + White Bar, FML.

To assess damage to the ocular surface induced by BAC and KIOM-2015E, we performed a TUNEL assay (Figure 3). After KIOM-2015E treatment for 2 weeks, eye balls were harvested, fixed, and paraffin-embedded. Cornea tissue section sections were prepared, deparaffinized, rehydrated with ethanol, and a TUNEL assay was performed using an in-situ cell death detection kit. TUNEL-positive cells were imaged by fluorescence microscopy. As shown in Figure 3A,B, 0.2% BAC strongly induced apoptosis in the corneal epithelium, stroma, and endothelium. The TUNEL-positive cell numbers were 0.17% ± 0.083% in the normal group compared with 20.06% ± 1.02% for the BAC-treated group. In the PBS treated group, TUNEL-positive cell numbers were reduced to 8.22% ± 0.80%. The results were not significantly different compared to those of oral administration (6.67% ± 3.98%). The effect of KIOM-2015E was increased for the extract prepared in 25% EtOH compared with the hot water extract (5.32% ± 1.03%). Furthermore, topical eye drops were superior to oral administration and dependent upon concentration (0.5 mg/mL; 3.37% ± 0.29%, 1 mg/mL; 3.10% ± 0.43%, respectively). These results suggest that KIOM-2015E was more effective for cell survival compared with commercial drugs (CsA; 3.29% ± 0.38%, FML; 3.78% ± 1.03%).

### 2.4. Histological Analysis of the Ocular Surface Following Treatment with Topical Eye Drops or Oral Administration of KIOM-2015E

**Figure 4:**
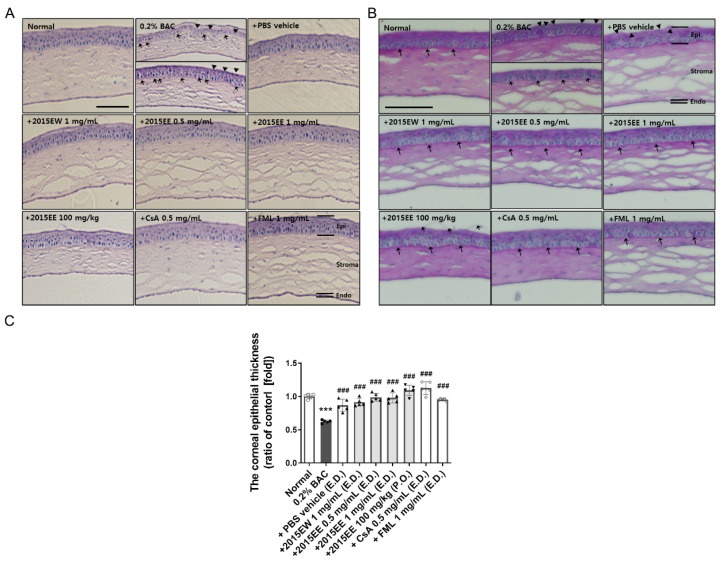
Histopathologic examination of the cornea after 2 weeks of topical or oral administration (n = 5/group) of KIOM-2015E. Alterations in the cornea were examined by H&E (**A**) and PAS (**B**) staining. An intact basement membrane was detected and the corneal epithelial thickness was measured in the BAC- or KIOM-2015E-treated corneas. (**A**) The cellular distribution and overall structure of the cornea were examined by H&E staining (arrow, Bowman’s layer; arrowheads, damaged epithelium). (**B**) PAS staining was carried out to detect the intact basement membrane (arrow, Bowman’s layer; arrowheads, damaged epithelium). (**C**) To quantify the results of corneal epithelial thickness, bar charts for the relevant images are shown. All experiments were performed in triplicate. The results in the bar graph are the average of three independent experiments. *** p < 0.001 ver. healthy control and *^###^ p* < 0.001 ver. 0.2% BAC. E.D.; topical eye drop, P.O.; oral administration, 2015EW; KIOM-2015EW (hot water extraction), 2015EE; KIOM-2015EE (25% EtOH extraction), CsA; cyclosporine, FML; Fluorometholone. Scale bar, 100 µm. Endo, endothelium; Epi, epithelium; Stroma, corneal stroma. White circle + white bar, Normal; dark gray circle + dark gray bar, 0.2% BAC; Dark gray triangle + white bar, PBS vehicle; Dark gray triangle + light gray bar, 2015EW and 2015EE eye drop; Dark gray inverted triangle + light gray bar, oral administration of 2015EE; White diamond + white bar, CsA; White Gakjinwon + White Bar, FML.

To evaluate corneal damage by BAC and the effects of KIOM-2015E, we performed immunohistochemistry using H&E and PAS staining (Figure 4). After treatment with KIOM-2015E for 2 weeks, the eye balls were harvested, fixed, and paraffin-embedded. Cornea tissue sections were prepared, deparaffinized, rehydrated with ethanol, and the specimens were stained with H&E or PAS.

Morphological changes were observed in the sectioned corneal tissue. No apparent structural abnormalities were detected in the mouse corneas from the normal controls, whereas the corneas exposed to 0.2% BAC for 2 weeks showed severe structural damage, including surface desquamation, an irregular surface, cell border loss, anisocytosis, and stromal shrinkage (Figure 4A). In BAC-induced dry eye mouse corneas, detached apical cells were more frequently observed. The basal epithelial cells in the BAC-treated group were spherical (Figure 4A, shot arrow) compared with columnar in the normal, KIOM-2015E-treated, and positive drug-treated corneas. The most superficial cells were loosely attached or slightly ragged in the BAC-treated corneas (Figure 4A,B, arrowheads). Furthermore, histological examination revealed an increased number of epithelial cell layers and considerably enlarged basal epithelial cells in the KIOM-2015E-treated compared with the BAC-treated group. Interestingly, irregular cells were still observed following the oral administration of KIOM-2015EE, but the cells were eliminated from the surface compared with that of the KIOM-2015E-treated group (Figure 4A,B). Examination of the PAS-stained sections revealed an intact basement membrane in the normal, KIOM-2015E-treated, and two positive drug-treated groups compared with the BAC-treated group, in which the basement membrane was reduced or lost and the corneal surface was rough or irregular (Figure 4B).

Chronic tear loss causes corneal epithelial thinning in dry eye animal models [23,24]. Therefore, we determined whether corneal epithelial thickness was altered by BAC or KIOM-2015E treatment. Epithelial layer thickness measurements were performed in all groups using ImageJ software (five mice per group, five measurements per cornea). As shown in Figure 4C and Supplementary Figure S4E, the thickness of the corneal epithelium significantly decreased in the BAC-treated group, whereas the corneal thickness of KIOM-2015E-treated mice was increased.

### 2.5. KIOM-2015E Suppresses BAC-Induced NF-κB Activation through p65 Nuclear Translocation Blockage in a Dry Eye Mouse Model

**Figure 5:**
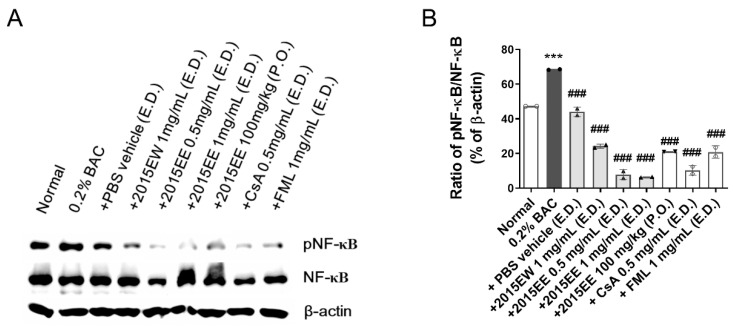
Western blot analysis of NF-κB activation in normal control, BAC-, PBS-, 1 mg/mL KIOM-2015EW, 0.5, 1 mg/mL KIOM-2015EE, 100 mg/kg KIOM-2015EE, 0.5 mg/mL CsA and 1 mg/mL FML-treated groups on day 14 after BAC-induced dry eye for 2 weeks. (**A**) Cornea whole tissue proteins were subjected to western blot analysis with the indicated antibodies. β-Actin was used as the internal control for total protein. (**B**) The bar charts represent the ratio of densitometric values relative to β-actin. All experiments were performed in triplicate. The results in the bar graph are the average of three independent experiments. *** p < 0.001 ver. healthy control and *^###^ p* < 0.001 ver. 0.2% BAC. E.D.; topical eye drop, P.O.; oral administration, 2015EW; KIOM-2015EW (hot water extraction), 2015EE; KIOM-2015EE (25% EtOH extraction), CsA; cyclosporine, FML; Fluorometholone. White circle + white bar, Normal; dark gray circle + dark gray bar, 0.2% BAC; Dark gray triangle + white bar, PBS vehicle; Dark gray triangle + light gray bar, 2015EW and 2015EE eye drop; Dark gray inverted triangle + light gray bar, oral administration of 2015EE; White diamond + white bar, CsA; White Gakjinwon + White Bar, FML.

After the application of KIOM-2015E for 2 weeks, the eyeballs were harvested. The cornea was selectively separated and protein extracts were prepared using a nuclear and cytoplasmic extraction kit. To determine whether the effect of KIOM-2015E was associated with the inhibition of BAC-induced NF-κB activation, NF-κB translocation was evaluated by western blot analysis. As shown in Figure 5, in whole lysates from cornea treated with 0.2% BAC, NF-kB p65 increased to 68.39% ± 0.22% compared with the normal control (47.25% ± 0.02%). However, following treatment with KIOM-2015E, p65 nuclear translocation was reduced from 24.23% to 6.34% (*p* < 0.001). Oral administration (20.97% ± 0.06%) also exhibited a similar result compared to the two positive drugs (CsA; 10.28% ± 2.89%, FML; 20.86% ± 3.62%). These results indicate that KIOM-2015E administration significantly inhibits the activation of NF-kB.

### 2.6. HPLC Analysis of the Marker Constituents in KIOM-2015E Extracts

**Figure 6:**
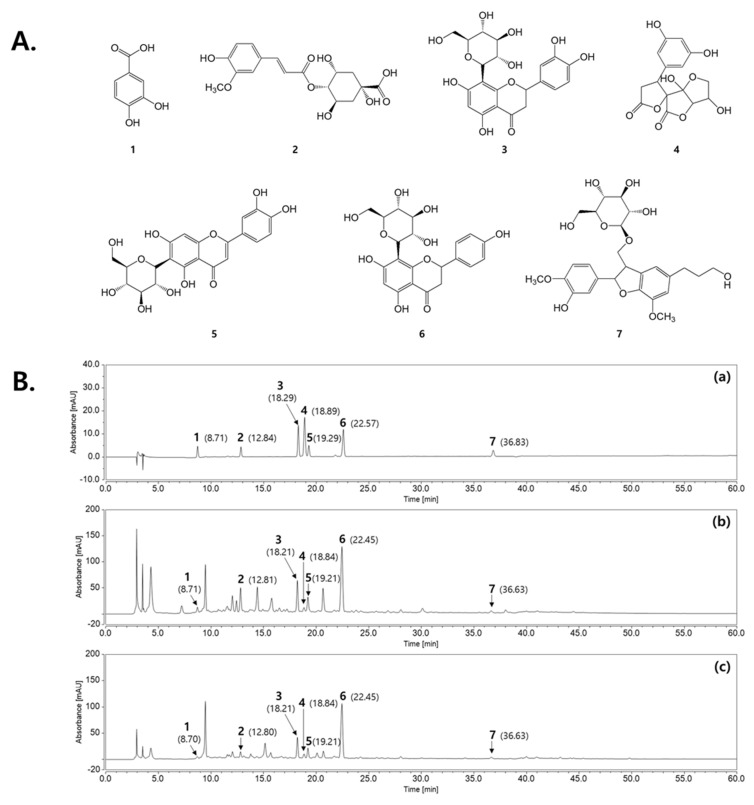
HPLC-DAD analysis of seven constituents in 2015EW and 2015EE. (**A**) Chemical structures of seven constituents: **1**, protocatechuic acid; **2**, 4-(E)-fruloyl quinic acid; **3**, orientin; **4**, dihydro-2′H,3H-spiro[furan-2,3′-furo[3,2-b]furan]-2′,5(3a′H,4H,5′H)-dione; **5**, isoorientin; **6**, vitexin; **7**, (7*S*,8*R*)-dihydrodehydrodiconiferylalcohol-9-ß-D-glucopyranoside. (**B**) HPLC–DAD analyses of seven constituents in 2015EW and 2015EE at 280 nm: (**a**) mixed marker constituents, (**b**) 2015EW, and (**c**) 2015EE. 2015EW, hot water extraction of KIOM-2015E; 2015EE, 25% ethanol extraction of KIOM-2015E.

Seven marker constituents, including protocatechuic acid (**1**), 4-(E)-fruloyl quinic acid (**2**), orientin (**3**), dihydro-2′H,3H-spiro[furan-2,3′-furo [3,2-b]furan]-2′,5(3a′H,4H,5′H)-dione (**4**), isoorientin (**5**), vitexin (**6**), and (7*S*,8*R*)-dihydrodehydrodiconiferylalcohol-9-ß-D-glucopyranoside (**7**) were detected at 280 nm based on the stability and maximum absorption rates of these compounds at baseline (**1**, 260 nm; **2**, 241 nm; **3**, 269 nm; **4**, 225 nm; **5**, 256 nm; **6**, 269 nm; **7**, 282 nm) (Figure 6A). As shown in Figure 6B, by comparing the retention times and UV spectral data with the marker constituents, the peaks from **1** to **7** in the chromatogram for the KIOM-2015E water extract (KIOM-2015EW) and the ethanol extract (KIOM-2015EE) were identified, respectively. The mixed marker constituents exhibited respective retention times of 8.71 min (**1**), 12.84 min (**2**), 18.29 min (**3**), 18.89 min (**4**), 19.29 min (**5**), 22.57 min (**6**), and 36.83 min (**7**) in the chromatogram. Under the same conditions, the retention times were 8.71 min (**1**), 12.81 min (**2**), 18.21 min (**3**), 18.84 min (**4**), 19.21 min (**5**), 22.45 min (**6**), and 36.63 min (**7**) for KIOM- 2015EW. These constituents were identified in KIOM-2015EE at similar retention times (**1**, 8.70 min; **2**, 12.80 min; **3**, 18.21 min; **4**, 18.84 min; **5**, 19.21 min; **6**, 22.45 min; **7**, 36.63 min).

## 3. Discussion

Dry eye is a common ocular surface disease with various causes. The BAC-induced dry eye mouse model established in this study shares the same pathologic changes with human dry eye, including epithelial apoptosis, goblet cell loss, tear film defect, and the inflammation, which have been identified in previous reports [8,9]. Inflammation is a key mechanism in the development of dry eye [25]. Anti-inflammatory therapy is administered to dry eye patients, particularly if inflammatory signs and irritation symptoms are observed [26].

Anti-inflammatory and immunosuppressive drugs are used to control the inflammatory cascade underlying these diseases. In particular, corticosteroids (FML) and CsA are the selective treatments for the more severe forms of dry eye disease. Nevertheless, extended topical corticosteroid application produces ocular long-term side effects [27,28], whereas CsA is not available in all countries as its use has been approved worldwide [29]. Therefore, it is necessary to develop new ocular therapeutic alternatives for the treatment of chronic or severe inflammatory ocular surface diseases.

Recently, we confirmed that KIOM-2015EW, but not KIOM-2015EE, has anti-inflammatory and anti-apoptotic effects in a hyperosmolar stress-induced in vitro dry eye model [22]. Three main compounds (orientin, isoorientin, and vitexin) were identified by HPLC-DAD analysis of KIOM-2015EW extracts, and each compound exhibited anti-inflammatory activity in a dose-dependent manner in hyperosmolar stress-induced human corneal epithelial cells. Orientin, isoorientin, and vitexin may be found in a variety of plant extracts and they have been shown to have anti-inflammatory effects in other studies [30,31,32].

In the present study, we demonstrated the anti-inflammatory effects of KIOM-2015E on the ocular surface of a BAC-induced dry eye mouse model. KIOM-2015E exhibited a higher potency compared with CsA or FML with respect to immunosuppression and corneal epithelial cell survival (Figure 2, Figure 3 and Figure 4); however, KIOM-2015E (100 mg/mL) oral administration was more effective than PBS, CsA, and FML, but less effective than topical administration. The effective concentrations of KIOM-2015E for the treatment of dry eye were 0.5 mg/mL and 1 mg/mL, whereas the effective concentrations of CsA and FML for the treatment of dry eye were 0.5 mg/mL and 1 mg/mL. Although this experiment was conducted at similar concentrations, the results demonstrated that KIOM-2015E had a superior effect.

As shown in previous reports, tears are hyperosmotic in dry eyes [33,34]. This state of tear hyperosmolarity activates mitogen-activated protein kinase (MAPK) cascades in corneal and conjunctival epithelial cells [3,35,36]. In our previous in vitro study, we demonstrated that KIOM-2015EW regulates MAPK signaling [22]. The activated kinases initiate a cascade of protein phosphorylation involving multiple kinases and activate nuclear transcription factors, such as NF-KB, e.g., that the expression levels of TNF-α, IL-1β, and IL-6 were downregulated in BAC-induced dry eye after treatment with KIOM-2015E. The phosphorylation of NF-KB was reduced along with a decrease in TNF-α, IL-1β, and IL-6 in BAC-induced dry eye mice following treatment with KIOM-2015E. Yeh et al. demonstrated that pro-inflammatory cytokines, such as TNF-α, IL-1, increase apoptosis of ocular surface epithelial cells in a dry eyes and the punctate epithelial erosion that is observed in the cornea and conjunctiva of patients with dry eye disease may be attributed to the sloughing of apoptotic epithelial cells [37]. Consistent with our study, we observed an increase in epithelial cell apoptosis in the dry eye-induced cornea. Using a TUNEL assay, we determined the effect of KIOM-2015E on the prevention of dry eye and/or BAC-induced epithelial cell apoptosis (Figure 3). Topical KIOM-2015EE (0.5 and 1 mg/mL) and KIOM-2015EW (1 mg/mL) exhibited no toxicity and significantly reduced epithelial cell apoptosis; however, oral KIOM-2015EE (100 mg/kg) was not significantly different from PBS treatment of dry eyes. The Lissamine Green B staining score was also similar to the results of the TUNEL assay (Figure 1 and Figure 3). Although drug efficacy was reduced in the case of oral administration, it was still more effective than CsA and FML for tear production (Figure 1) and corneal epithelial thickness (Figure 4).

In summary, the anti-inflammatory effects of topical or oral KIOM-2015E on dry eye have not been clearly established. Our study systematically examined the effects of topical or oral KIOM-2015E on BAC-induced dry eye for the first time and suggested that KIOM-2015E effectively improves the clinical features, including tear production, Lissamine Green B staining score, and the inflammatory index. It was also capable of maintaining corneal epithelial integrity and thickness and inhibiting corneal inflammation. Therefore, KIOM-2015E has potential as a topical, as well as oral, anti-inflammatory drug for the treatment of dry eye.

## 4. Materials and Methods

### 4.1. Materials

BAC and lissamine green B were purchased from Sigma (Sigma-Aldrich, St. Louis, MO, USA). Anti-tumor necrosis factor (TNF)-α, anti-interleukin (IL)-6, and anti-IL-1β were purchased from Abcam (Cambridge, MA, USA). VECTASHIELD^®^ Mounting Medium with DAPI was purchased from Vector Laboratories (Burlingame, CA, USA). An in situ cell death detection kit for terminal deoxynucleotidyl transferase dUTP nick end labeling (TUNEL) assay was purchased from Roche (In Situ Cell Death Detection Kit, Fluorescein; Roche Diagnostics, Indianapolis, IN, USA). Anti-β-actin was purchased from Santa Cruz Biotechnology Inc. (Santa Cruz, CA, USA). NF-κB and p-NF-κB were purchased from Cell Signaling Technology (Danvers, MA, USA). CsA and FML were obtained from Hanlim (Tsporin eye drops 0.05% CsA; Fumelone eye drops 0.6 mL; Hanlim Pharm. Co., Seoul, South Korea).

### 4.2. Animals and Procedures

Seventy-eight 10-week-old female C57BL/6 mice (weight, 18–20 g; purchased from Samtako (Osan, Korea) were used in this study. The experiments were performed according to the guidelines of the Animal Care and Use Committee at KIOM and the Association for Research in Vision and Ophthalmology Statement for the Use of Animals in Ophthalmic and Vision Research. All animal experiments were approved by the Animal Care and Use Committee of the KIOM (Daejeon, Korea) with reference number D-16-014. On the basis of clinical evaluations (described below), the mice were randomly divided into 9 groups (Supplementary Figure S1): normal saline- (control) (n = 11), 0.2% BAC- (n = 11), PBS vehicle- (n = 8), KIOM-2015EW 1 mg/mL eye drop- (n = 8), KIOM-2015EE 0.5 and 1 mg/mL eye drop- (n = 8), KIOM-2015EE 100 mg/kg oral administration- (n = 8), and 2 positive (CsA and FML) eye drop- treated groups (n = 8). For the first 2 weeks, dry eye was induced in the mice by topical administration of 0.2% BAC [9]. The right eyes of 67 randomly chosen mice were treated twice daily (9 AM and 9 PM) topically with 5 µL of 0.2% BAC (BAC-treated group), whereas the other 11 mice were not treated (control group). BAC powder was dissolved in normal saline to prepare a 0.2% BAC solution. Over the next 2 weeks, the BAC-treated 67 mice were randomly divided into 8 groups. Five microliters of drugs (0.2% BAC- (n = 11), PBS vehicle- (n = 8), KIOM-2015EW 1 mg/mL eye drop- (n = 8), KIOM-2015EE 0.5 and 1 mg/mL eye drop- (n = 8), KIOM-2015EE 100mg/kg oral administration- (n = 8), and 2 positive (CsA and FML) eye drop- treated groups (n = 8)) were administered to the mice in the 8 groups. The right eyes of all the mice were treated thrice daily (9 AM, 3 PM, and 9 PM) with topical drops and once a day (3 PM) orally. During the treatment, clinical evaluations were performed by a single masked ophthalmologist. On day 30, all the mice were anesthetized. Eye tissues were harvested carefully for histological analysis after applying the methods described below.

### 4.3. Lissamine Green B Staining and Quantification

After anesthesia with isoflurane, 5 μL of 1% lissamine green B (Lissamine™ Green B, Sigma-Aldrich, St. Louis, MO, USA, Cat#199583) was applied to the ocular surface. Photographs of the eye were taken using a digital camera (Nikon Corp., Tokyo, Japan) fitted to a manual surgical microscope (Leica M651; Leica Microsystems GmbH, Wetzlar, Germany). The scoring index was analyzed as previously described [38]. Blind was assessed by four observers, and the average score of all was used for each eye. The difference in the staining score at the end of week 4 with respect to the pretreatment baseline was reported.

### 4.4. Paraffin Embedding and Histological Examination

Corneal tissues were isolated from the harvested eye tissues. The slide preparation process was described in a previous study [39]. Images were captured using the software NIS-Elements BR 4.50 of Nikon fluorescence microscope (Nikon, Tokyo, Japan).

### 4.5. Measurement of Tear Volume

Tear production was measured by the phenol red thread tear test using cotton threads (Zone-Quick; Yokota, Tokyo, Japan) [40] at a similar time point (3 PM) on days 0, 1, 4, 7, and 14D in the standard environment. Mice were kept immobile by isoflurane. The lower eyelid was pulled down slightly, and a 1 mm portion of the thread was placed on the palpebral conjunctiva for 60 s at a specified point approximately one-third of the distance from the lateral canthus of the lower eyelid. The red portion of the thread is measured in millimeters.

### 4.6. PAS Staining

Sections were stained with periodic acid-Schiff (PAS) reagent (Sigma-Aldrich, St. Louis, MO). Three sections were examined for each cornea, and images were captured at equidistant intervals from the corneal mid-point with a DS-Fil digital camera (Nikon, Tokyo, Japan) attached to a Nikon microscope.

### 4.7. TUNEL Assay

The specimens were subjected to TUNEL assay using an in situ cell death detection kit, fluorescein (Roche Diagnostics, Cat. No. 11 684 795 910), by the manufacturer’s instructions and previous research [39]. The positive control was incubated with deoxyribonuclease I (3000 U/mL in 50 mM Tris/HCl), and the negative-control sample was incubated with label-solution only. TUNEL-positive cells were viewed by the software NIS-Elements BR 4.50 of Nikon fluorescence microscope (Nikon, Tokyo, Japan).

### 4.8. Immunofluorescence Staining

For immunofluorescence staining, sectioned corneal tissue slides were deparaffinized in xylene and then rehydrated through an ethanol series. Staining was performed as described in detail before [39]. The specimens were then incubated with monoclonal or polyclonal antibodies against TNF-a, IL-1β, and IL-6 (diluted 1:50–100).

### 4.9. RNA Isolation and Real-Time Reverse Transcription-Polymerase Chain Reaction Analysis

After isolating the corneal tissues from the harvested eye tissues, total RNA was isolated from cornea tissue using the RNA-spin total RNA extraction kit (iNtRoN, Daejeon, Korea) according to the manufacturer’s instructions. Reverse transcription was carried out in a 20 μL reaction with 0.5 μg of total RNA transformed into cDNA using AccuPower CycleScript RT premix (Bioneer, Daejeon, Korea). For measurements of TNF-α, IL-1β, IL-6, and GAPDH mRNA, the PCR conditions were as follows: 12 cycles of primer annealing at 30 °C for 30 s, cDNA synthesis at 45 °C for 4 min, melting of the secondary structure and cDNA synthesis at 55 °C for 30 s, and heat inactivation at 95 °C for 5 min. The PCR-amplified primers used in this study are described in Table 1. Gene expression was quantified by real-time PCR using the AccuPower 2× Greenstar qPCR Master (Bioneer) according to the following protocol: pre-denaturation at 95 °C for 10 min, followed by 40 cycles of 95 °C for 10 s, 60 °C for 30 s, and 72 °C for 30 s. Amplifications were carried out using QuantStudio 6 (Thermo Fisher Scientific, MA, USA), The fold change in the expression of the target gene relative to the control was normalized to GAPDH using the 2^−ΔΔCt^ method.

### 4.10. Preparation of Cellular Protein Extraction and Western Blot Analysis

Western blotting was performed using standard techniques as in our previous research (NE-PER Nuclear and Cytoplasmic extraction reagents, Thermo-Scientific, Cat#78835) [41]. An equal amount of protein lysates (30 μg) in RIPA buffer (Millipore Corporation, Billerica, MA, USA) by adding protease inhibitor cocktail and phosphatase inhibitors (Roche Diagnostics, Basel, Switzerland). Nitrocellulose membranes were then placed immediately into a blocking solution (5% non-fat milk) at room temperature for 1 h, and incubated with the following diluted primary antibodies; NF-κB (1:1000, Cell Signaling Technologies, Catalog No. 6956), pNF-κB (1:500, Cell Signaling Technologies, Catalog No. 3039), and β-actin (1:1000, Santa Cruz Biotechnology, Catalog No. sc47778) in TBS-T buffer (Tris–HCl based buffer containing 0.2% Tween 20, pH 7.5). Horseradish-conjugated secondary antibody labeling was detected by enhanced chemiluminescence and blots were exposed to radiographic film. Band intensities were measured using ImageJ software (US National Institutes of Health, Bethesda, MD, USA).

### 4.11. Preparation of Standard Compound Solutions and Samples for HPLC Analysis

As marker constituents of KIOM-2015E, the seven kinds of single compounds, including protocatechuic acid (**1**), 4-(E)-fruloyl quinic acid (**2**), orientin (**3**), dihydro-2′H,3H-spiro[furan-2,3′-furo[3,2-b]furan]-2′,5(3a′H,4H,5′H)-dione (**4**), isoorientin (**5**), vitexin (**6**), and (7*S*,8*R*)-dihydrodehydrodiconiferylalcohol-9-ß-D-glucopyranoside (**7**), were obtained from Professor Byung Sun Min in Daegu Catholic University (Gyeongsan-si, Korea). The stock solutions of marker constituents were prepared in 100% methanol at 1 mg/mL, which were diluted and mixed to a concentration of 12 ppm, respectively. KIOM-2015EW and KIOM-2015EE were respectively extracted in 100% methanol by sonication for 30 min, and prepared at 10 mg/mL. All working solutions analyzed in this study were filtered through a 0.2 μm syringe membrane filter from Whatman Ltd. (Maidstone, UK) for HPLC analysis.

### 4.12. Chromatographic Conditions

The KIOM-2015E samples and marker constituents were analyzed using Dionex Ultimate 3000 HPLC system (Dionex Co., Sunnyvale, CA, USA), equipped with a binary pump, an auto-sampler, a column oven and a diode array UV/VIS detector (DAD). The analysis of output signals from detector was performed by Chromeleon software. All chromatographic separations were performed on a Xbridge^®^ C18 column (5 μm, 4.6 × 250 mm, Waters Co., Milford, MA, USA) in column temperature maintained at 40 °C. The mobile phase was consisted of water with 0.1% trifluoroacetic acid (TFA; Sigma-Aldrichi, St. Louis, MO, USA) (A) and acetonitrile (B) with gradient elution at a flow rate of 1 mL/min. All working solutions were injected 5 µL. The HPLC elution condition was optimized as follows: 5% B at 0–3 min; 5–10% B at 3–5 min; 10–15% B at 5–15 min; 15–22% B at 15–35 min; 25% B at 35 min, 25–45% B at 35–55 min; 45–70% B at 55–60 min. UV absorption was monitored at 280 nm and the total run time was 60 min.

### 4.13. Statistical analysis

Data were evaluated with one-way ANOVA by Turkey’s test. The analyses were performed using GraphPad PRISM software^®^ (GraphPad PRISM software Inc., Version 5.02, CA, USA). Results are expressed as means ± standard error of the mean (SEM), and *p*-values <0.05 were considered as significant.

## 5. Conclusions

Taken together, the results of this study indicate that dry eye syndrome treatment strategies should include strategies to protect the eyes from inflammatory damage by regulating inflammatory factors in addition to mediating tear secretion-related phenomena. Application of KIOM-2015E showed clinical improvements and inhibited the inflammatory response, alleviating the signs of dry eye. The results indicated that KIOM-2015E has potential as a therapeutic agent in the clinical treatment of dry eye. Therefore, the development of products such as KIOM-2015E derived from natural products for the treatment of corneal cell damage and inflammatory damage caused by dry eye syndrome will improve the quality of life of patients who have difficulty in daily living due to dry eye syndrome.

## Figures and Tables

**Table 1:** Primers used for qPCR.

Gene Name	NCBI Accession No.	Primer Sequences	
**TNF-α**	M13049.1	F	5′-GGTTCTGTCCCTTTCACTCA-3′
		R	5′-CCTCTTCTGCCAGTTCCA-3′
**IL-1β**	M15131.1	F	5′-CCTCACAAGCAGAGCACAA-3′
		R	5′-AGAAACAGTCCAGCCCATAC-3′
**IL-6**	DQ788722.1	F	5′-CTCTGGGAAATCGTGGAAAT-3′
		R	5′-CCAGTTTGGTAGCATCCATC-3′
**GAPDH**	GU214026.1	F	5′-CTGCTCCTCCCTGTTCCA-3′
		R	5′-CACACCGACCTTCACCAT-3′

## Data Availability

The data used to support the findings of this study are available from the corresponding author upon request.

## Supplementary Materials

The following supporting information can be downloaded at: https://www.mdpi.com/article/10.3390/ijms232314964/s1.
