# Supplementary material for: Therapeutic Effects of Acer palmatum Thumb. Leaf Extract (KIOM-2015E) on Benzalkonium Chloride-Induced Dry Eye in a Mouse Model
Source: Int J Mol Sci. 2022 Nov 29;23(23):14964. doi: 10.3390/ijms232314964 (PMC9736398; doi:10.3390/ijms232314964)
Supplement: Supplementary file 1 [file ijms-23-14964-s001.zip › ijms-2026390-supplementary.pdf]

Supplementary Information

Therapeutic Effects of *Acer Palmatum* Thumb. leave extract (KIOM-2015E) on Benzalkonium Chloride-Induced Dry Eye in a Mouse Model

Nam-Hui Yim[1,†], Eunhee Park[2,†], Won Kyung Cho[1], Yeoun-Hee Kim[3,*], Jin Yeul Ma[1,*]

[1] Korean Medicine (KM)-Application Center, Korea Institute of Oriental Medicine (KIOM), 70, Cheomdan-ro, Dong-gu, Daegu 41062, Republic of Korea
[2] Helixmith, 21, Magokjungang 8-ro 7-gil, Gangseo-gu, Seoul 07794, Republic of Korea
[3] R&D Center, Etnova Therapeutics Corp., 198, Saneop-ro, Gwonseon-gu, Suwon-si 13207, Republic of Korea
† Nam-Hui Yim and Eunhee Park contributed equally to this work
* Correspondences: yhkim@etnova.co.kr (Y.-H.K.); jyma@kiom.re.kr (J.Y.M.); Tel.: +82-31-776-3403 (Y.-H.K.); +82-53-940-3812 (J.Y.M.)

Supplementary Figure S1. Schematic of experimental design
Supplementary Figure S2. Changes in ocular surface at 2 weeks and 4 weeks in 0.2% BAC treatment.
Supplementary Figure S3. Changes in ocular surface at 2 weeks and 4 weeks in 0.2% BAC treatment.
Supplementary Figure S4. Source data of western blot analysis shown in Figure 2A and 2B
Supplementary Figure S5. Quantification tables.

Figure S1

(A)

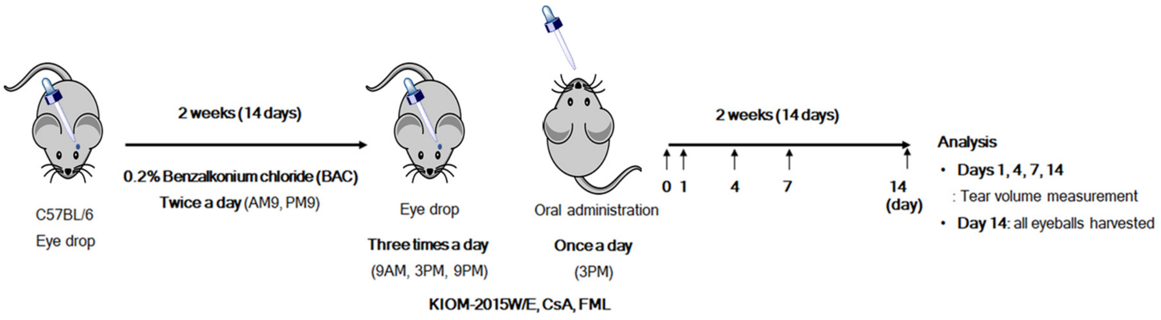

(B)

Conditions (groups)	1 week	2 week	3 week	4 week
Normal (n = 11)	Non-treatment			
0.2% BAC only (n =11)	0.2% BAC		0.2% BAC	
0.2% BAC + PBS vehicle (E.D.) (n = 8)	0.2% BAC		E.D. Normal saline	
0.2% BAC + KIOM-2015EW1 mg/ml (E.D.) (n = 8)	0.2% BAC		E.D. KIOM-2015EW	
0.2% BAC + KIOM-2015EE 0.5 mg/ml (E.D.) (n = 8)	0.2% BAC		E.D. KIOM-2015EE	
0.2% BAC + KIOM-2015EE 1 mg/ml (E.D.) (n = 8)	0.2% BAC		E.D. KIOM-2015EE	
0.2% BAC + KIOM-2015EE 100 mg/kg (P.O.) (n = 8)	0.2% BAC		P.O. KIOM-2015EE	
0.2% BAC + CsA 0.5mg/ml (E.D.) (n = 8)	0.2% BAC		E.D. T-sporin	
0.2% BAC + FML 1mg/ml (E.D.) (n = 8)	0.2% BAC		E.D. Fumelone	

0.2% BAC : twice a day (AM 9:00, PM 9:00), 5 μl/eye rop

Drug treatment : three times a day (AM 9:00, PM 1:00, PM9:00), 5 μl/eye drop

once in a day (PM 1:00), 100 μl/oral administration

Supplementary Figure S1. Schematic of experimental design. Schematic of the experimental protocol of benzalkonium chloride (BAC)-induced DED model and drug application (A), and tabulated (B).

Figure S2

(A)

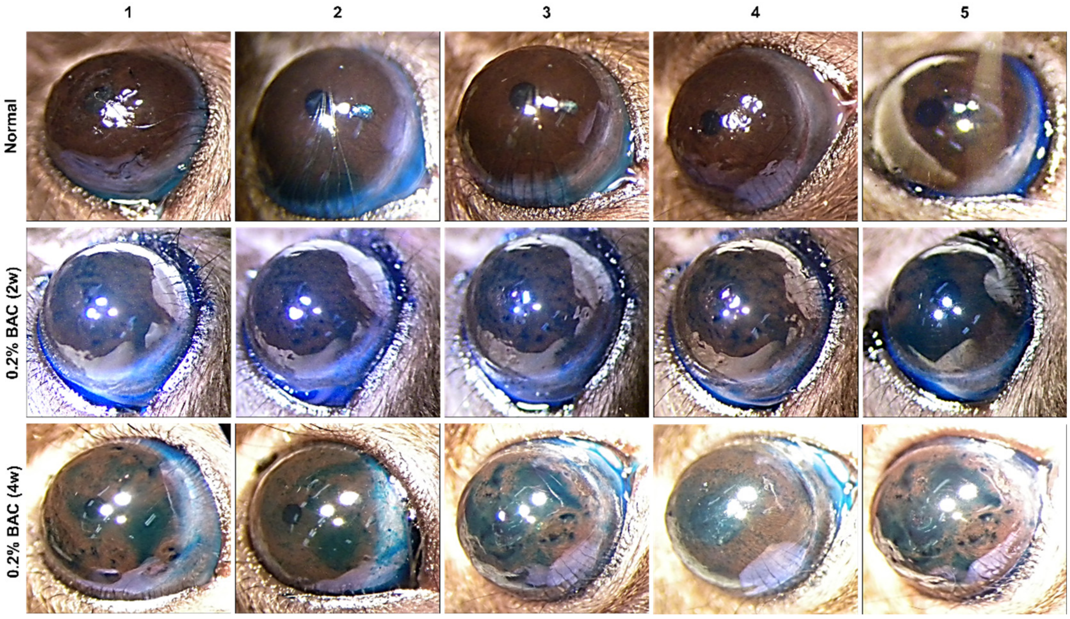

(B)

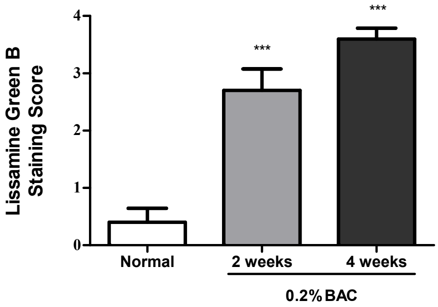

Supplementary Figure S2. Changes in ocular surface at 2 weeks and 4 weeks in 0.2% BAC treatment. (A) Representative images of corneal epithelium integrity including lissamine green B staining at 2 and 4 weeks of BAC treatment (n = 5). (B) Change in lissamine green B staining score relative to treatment level in BAC treatment. Statistical significance was analyzed by performing one-way ANOVA with Turkey's test using GraphPad Prism. Differences were considered statistically significant as follows: ***P<0.001 vs. Normal. BAC; benzalkonium chloride, 2w; 2 week, 4w; 4 week.

Figure S3

(A)

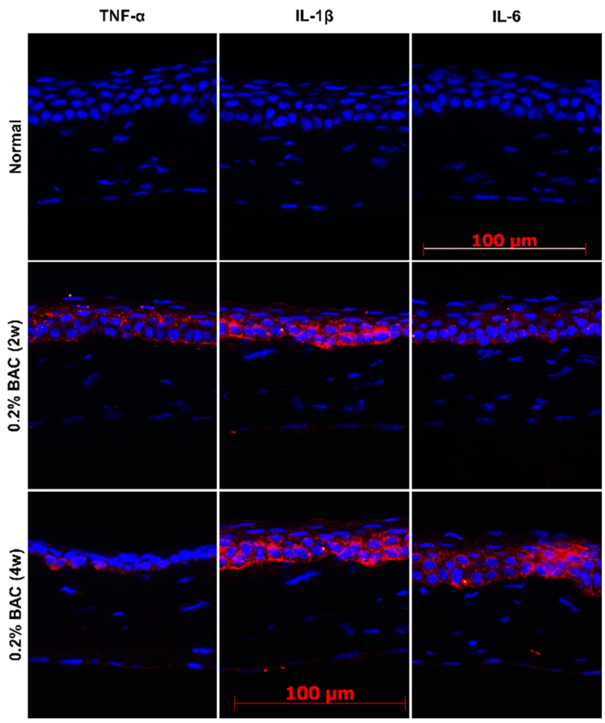

(B)

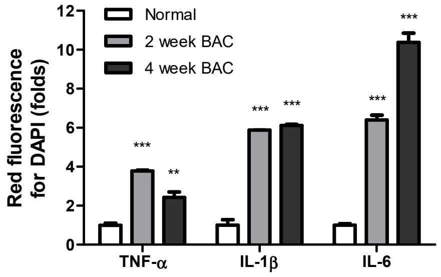

Supplementary Figure S3. Changes in ocular surface at 2 weeks and 4 weeks in 0.2% BAC treatment. (A) Representative images for corneal cytokines immunofluorescence, merged with DAPI (n = 3). Scale bar, 100 µm. (B) The density of cells positively stained for fluorescence staining in the cornea tissues. Quantification of red-fluorescence density was performed using the ImageJ software program. These experiments were performed in triplicate, and the results are the average of 3 independent experiments. The results of the bar graph are the averages of 3 independent experiments. Statistical significance was analyzed by performing one-way ANOVA

with Turkey's test using GraphPad Prism. Differences were considered statistically significant as follows: ***$P<0.001$, **$P<0.01$ vs. Normal. BAC; benzalkonium chloride.

Figure S4. Source data of western blot analysis shown in Figure 5A.

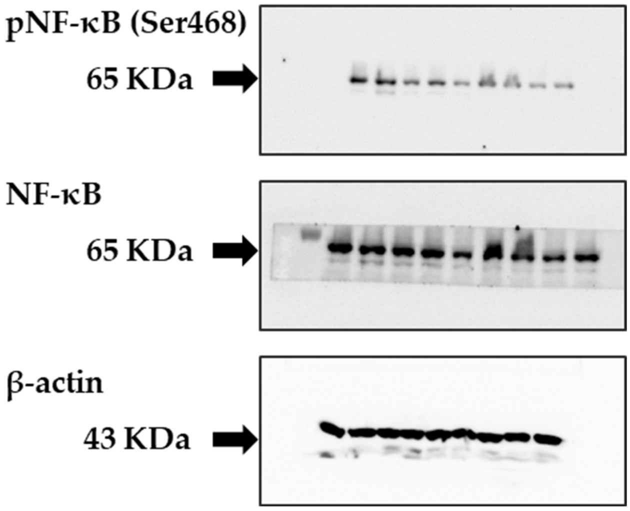

Figure S5

(A)

Tear production (mm)									
**Day**	**Normal**	**0.2% BAC**	**+ PBS vehicle**	**+ EW 1 mg/mL**	**+ EE 0.5 mg/mL**	**+ EE 1 mg/mL**	**+ EE 100 mg/kg**	**+ CsA 0.5 mg/mL**	**+ FML 1 mg/mL**
**Mean±SD**									
**0D**	4.50±0.51	2.61±0.76	-	-	-	-	-	-	-
**1D**	4.42±1.85	2.13±1.26	2.33±0.81	2.46±1.52	3.16±1.15	3.22±1.48	3.00±1.65	2.16±0.88	3.95±1.60
**4D**	4.54±2.61	2.25±0.91	2.69±1.27	6.07±2.39	7.76±0.74	7.56±1.55	5.84±1.80	3.26±1.41	6.83±1.52
**7D**	4.97±1.39	2.53±1.02	5.48±1.38	6.96±2.16	7.95±1.42	7.97±0.80	5.85±1.26	6.99±1.42	7.36±1.09
**14D**	5.21±0.92	2.57±0.52	6.69±1.79	8.25±2.82	10.62±1.46	10.33±2.43	8.08±1.57	6.47±1.26	7.46±2.09

(B)

Lissamine green B staining score			
**Group**	**Mean**	**Min**	**Max**
**Normal**	0.67	0	1
**0.2% BAC**	3.67	3	4
**+ PBS vehicle**	1.67	1	2
**+ EW 1 mg/mL**	1.00	1	1
**+ EE 0.5 mg/mL**	0.67	0	1
**+ EE 1 mg/mL**	0.33	0	1
**+ EE 100 mg/kg**	1.33	1	2
**+ CsA 0.5 mg/mL**	0.83	0.5	1
**+ FML 1 mg/mL**	0.67	0.5	1

(C)

**Groups**	**Fluorescence analysis**		
	**Intensity (mean±SD, %)**		
	**TNF-α**	**IL-1β**	**IL-6**
**Normal**	9.26±1.28	12.81±1.77	7.22±0.60
**0.2% BAC**	25.51±2.60	61.35±2.74	62.22±3.98
**+ PBS vehicle**	13.29±1.75	20.72±2.16	21.93±3.97
**+ EW 1 mg/mL**	12.32±0.59	20.25±1.27	16.94±0.36
**+ EE 0.5 mg/mL**	6.53±0.18	14.98±1.04	14.69±0.97
**+ EE 1 mg/mL**	5.60±1.00	15.43±1.78	11.00±0.90

**+ EE 100 mg/kg**	10.87±1.57	21.17±2.29	19.17±5.07
**+ CsA 0.5 mg/mL**	7.85±0.61	20.43±2.47	9.52±0.82
**+ FML 1 mg/mL**	7.20±1.16	21.28±2.31	13.35±2.29

(D)

**Groups**	**Relative mRNA expression analysis**		
	**Expression (mean±SD, fold)**		
	**TNF-α**	**IL-1β**	**IL-6**
**Normal**	1	1	1
**0.2% BAC**	1.11±0.05	1.12±0.01	1.14±0.05
**+ PBS vehicle**	0.99±0.00	1.10±0.01	1.10±0.01
**+ EW 1 mg/mL**	0.87±0.09	1.00±0.02	1.00±0.02
**+ EE 0.5 mg/mL**	0.84±0.05	0.97±0.00	0.97±0.01
**+ EE 1 mg/mL**	0.83±0.03	0.96±0.02	0.96±0.02
**+ EE 100 mg/kg**	0.95±0.05	0.98±0.04	0.98±0.04
**+ CsA 0.5 mg/mL**	0.87±0.09	0.97±0.03	0.97±0.03
**+ FML 1 mg/mL**	0.86±0.09	0.96±0.02	0.96±0.02

(E)

**The corneal epithelial thickness**									
**Groups**	**Normal**	**0.2% BAC**	**+ PBS vehicle**	**+ EW 1 mg/mL**	**+ EE 0.5 mg/mL**	**+ EE 1 mg/mL**	**+ EE 100 mg/kg**	**+ CsA 0.5 mg/mL**	**+ FML 1 mg/mL**
**Fold (mean±SD)**	1±0.03	0.62±0.02	0.87±0.09	0.91±0.06	0.99±0.06	0.98±0.07	1.09±0.08	1.13±0.10	0.95±0.02

Supplementary Figure S5. Quantification tables. (A) Tear production measurement with phenol-red cotton thread method. (B) The change of lissamine green B staining scores. (C) Fluorescence intensity analysis of immunofluorescence staining. (D) Analysis of TNF-α, IL-1β and IL-6 mRNA expression levels in BAC-induced dry eye mouse model by quantitative real time PCR (qRT-PCR). Average results of 3 independent experiments. Values are presented as mean±SD. (E) Analysis of corneal epithelial thickness in BAC-induced dry eye mouse model. Five photographs were randomly selected in each group, and the corneal thickness was measured using the ImageJ program (pixel value). And then the respective values were expressed as folds to normal, and the five values were averaged.
